# Targeted drugs and Psycho-oncological intervention for breast cancer patients

**DOI:** 10.1186/s12952-016-0049-9

**Published:** 2016-04-01

**Authors:** Flavio D’Abramo, Ute Goerling, Cecilia Guastadisegni

**Affiliations:** Department of Psychology, Freie Universität Berlin, Hittorfstr. 16, 14195 Berlin, Deutschland; Charité Comprehensive Cancer Center, Charitéplatz 1, 10117 Berlin, Deutschland; Department of Environment and Primary Prevention, Italian National Institute of Health, ISS, V.le Regina Elena 299, 00161 Rome, Italy

**Keywords:** Cancer targeted drugs, Breast cancer, Psycho-oncological intervention, Psychological intervention, Quality of life, Vascular endothelial growth factor (VEGF), Bevacizumab, Avastin, Segt

## Abstract

Personalized medicine is a new field based on molecular biology and genomics in which targeted tumor therapies are administered to patients. Psycho-oncology is a complementary approach that considers social and psychological aspects of patients as part of the treatments for cancer patients.

The aim of this mini-review is to weigh clinical benefits for breast cancer patients of both treatments and possibily enhance benefits by modulating the use of both interventions. We have compared and evaluated on the one hand the use of anti Vascular Endothelial Growth Factor and, on the other hand, psycho-oncological interventions in metastatic and non-metastatic breast cancer patients.

Both treatments did not increase survival of metastatic breast cancer patients, while in a selected study psycho-oncological interventions extended lifespan of non-metastatic breast cancer patients and ameliorate psychological and social factors of metastatic breast cancer patients. Because the two approaches address completely different aspects of cancer patients, if the comparison is limited to the extension of survival, the value of these two treatments cannot be assessed and compared.

It is likely that by comparing patients reported outcomes, possibly by using standardized Quality of Life questionnaires, both patients and health care providers can weigh the benefits of the two treatments. It is therefore important to evaluate the use of cancer patients’ quality of life measures as a mean to improve their experiences about life and treatment, and possibly to extend their survival.

## Introduction

Medicine considers the objective scientific knowledge of biology and physiology and, on the other hand, also the subjective personal knowledge derived from the persons involved in the medical processes [1]. We sought to dissect the dualistic epistemology of medicine that holds a dichotomy between pure objectivity and pure subjectivity by considering the possible links between molecular biology and psychology in the treatment of cancer patients. It is the synthesis of these far, often opposed disciplines that is central for developing a patient-centered medicine [2, 3]. As we show below, when molecular medicine and psychology are considered as complementary they can address care of patients in a more complex, articulated and effective manner.

One of the most promising frontiers of molecular medicine is represented by personalized medicine. Medicine is by definition, personalized, as physicians make a diagnosis and a prognosis using information obtained from a person’s individualized symptoms, physical characteristics, health and family history, habits and exposures [4]. This new discipline of personalized medicine differs from the old, traditional medicine mainly because it is pre-emptive, highlighting the intention to predict the effects of the targeted intervention and its benefits from the understanding of the molecular biology of cancer development [5]. In the case of the new personalized medicine, the use of targeted drugs is only limited to the subgroup of patients showing specific tumor genetic variation – i.e. molecular biomarkers – that are presumably associated with positive clinical outcomes. In the present study the choice of the anti Vascular Endothelial Growth Factor (VEGF) targeted drug bevacizumab, for which no molecular biomarkers have yet found was dictated by the influence that psychological interventions might have on the circulating level of this molecule (see below the ‘discussion’ section).

The focal point of personalized medicine as defined by the use of targeted drugs is the tumor itself, firstly evaluated by using the Progression Free Survival endpoint (PFS) and afterwards survival of patients measured through the Overall Survival endpoint (OS), that is often coupled with patients’ psychological status or toxicity of treatments and related problems. These latter aspects can be addressed through the asessement of patients’ Quality of Life (QoL). The oncologists who, instead of prescribing medications only, have also evaluated the psychological effects of the oncological diagnosis and interventions on survival and well-being of cancer patients have approached these patients’ needs.

Psycho-oncology is a discipline which started in the United States in mid-1970s [6] and it allows cancer patients to benefit from social and psychological programs that contribute restoring health through alleviating the stress caused by the diagnosis of cancer and related treatments. These psycho-oncological interventions ultimately ameliorate patients’ outcome [7]. The psycho-oncological approach for cancer patients was mainly developed in hospices for those patients who failed responding to chemotherapy and where the main focus is alleviation of symptoms. Indeed, in those cases where cancer patients are getting closer to the final days of their lives, an approach mainly based on drug therapies is very often detrimental to their QoL [8]. Psychosocial aspects have played a very minor role in mainstream oncology. The aim of this article is to complement the targeted drug bevacizumab with psycho-oncology interventions in breast cancer patients by reporting measures of survival of patients (OS) and other measures related to their psychological and social well-being.

## Methods

Both, bevacizumab and psycho-oncology have been scrutinized using clinical trials. We formulated two questions: (i) what is the clinical trial utilized to approve bevacizumab for metastatic breast cancer patients? And (ii) what are the clinical trails that have been evaluating the use of psycho-oncological interventions on breast cancer patients and related effects on patients’ survival? To address these questions we decided to query both, the Food and Drug Administration (FDA) web site and the European Medicines Agency (EMA) one to individuate the trials utilized for the approval of bevacizumab for first-line treatment of metastatic breast cancer patients [9]. To complete the ovierview on bevacizumab we selected, through a PubMed free search, two more trials evaluating its use in early-stage breast cancer patients.

To analyse the psychological interventions for breast cancer patients we developed a search algorithm, we used in PubMed, based on key words relevant for an evaluation of patients’ survival: (psycho-oncology [tw] OR psychologic intervention [tw] OR psychosocial intervention [tw] OR segt [tw] OR cegt [tw]) AND (breast cancer [tw]) AND (survival [tw]).

In a second step we defined criteria to determine the relevance of retrieved articles. These criteria were defined on the base of methodologies utilized in the articles. As inclusion criteria we decided to focus on (i) clinical trials measuring the effect of psychological interventions on overall survival of breast cancer patients. We excluded other studies than clinical trials, such as observational studies, cohort studies, case–control studies, meta-analysis, reviews and theoretical studies.

Moreover, we extended the search on articles contained in the bibliographic references of the selected articles, focusing on the original trials – for instance data published on the same trials. We also searched in PubMed for updates of those trials retrieved in the initial search. The search was limited to articles published untill September 15th 2015.

Besides the articles on the trials for bevacizumab and psychosocial intervention for breast cancer patients, we have been analyzing other theoretical articles on biological mechanisms and explanations that are at the base of the effects elicited by these two kinds of intervention. Furthermore, we included three meta-analysis scrutinizing the association between depression (and emotional distress) and survival of cancer patients. Additionally, as we found out the importance of endpoints other than survival, we focused also on quality of life (QoL) for breast cancer patients.

## Results

One clinical trial was selected through the FDA and EMA websites to show data utilized for the approval of bevacizumab. The trial of Miller et al. was used for the approval of bevacizumab for metastatic breast cancer patients [9]. We selected two more trials through a free PubMed search evaluating the use of bevacizumab for early-stage cancer patients [10, 11].

The search in PubMed about psychological interventions for breast cancer patients yielded 21 articles . We selected four clinical trials to evaluate the data for the use of psychosocial interventions on breast cancer patients. The first and the second we selected were done on breast cancer patients, by Spiegel et al. [12], and by Kissane et al. [13] respectively. The third and fourth selected studies were done on early-stage breast cancer patients, by Andersen et al. [14] and by Kissane et al. [15] respectively. For gathering quantitative data on psychological interventions for breast cancer patients we excluded those articles other than clinical trials [16–32]. The study by Spiegel et al. conducted in 1989 [12] was eventually replaced with the updated trial conducted by the same group in 2007 [33]. We lately recovered two articles initially excluded showing biological insights useful to interpret the data and develop further directions [16, 29]. Moreover, we included other two articles by Andersen et al. to complete the data on the trial we initially selected [34, 35]. The data on effects of bevacizumab and psychological interventions for metastatic and early-stage breast cancer patients are systematized in Table 1.

**Table 1:** Synthesis of the data reported in selected clinical trials. Terms in bold within the 'Intervention' column indicate the specific medical intervention/treatment assessed in the trial. Terms in bold within the 'Outcomes' column indicate the trial's endpoints

Study	Intervention	Patients group	Number of patients	Length of the study	Outcomes
Miller et al. 2007 [9]	Randomized, phase 3 trial.	Metastatic breast cancer	*N* = 722 (Control *N* = 354; Invervention *N* = 368)	2.5 years	**PFS**> 5.9 months (*p* < 0.001)
	Efficacy and safety on paclitaxel with or without **bevacizumab**				**OS** >1.5 months (*p* < 0.16)
Spiegel et al. 2007 [33]	Randomized prospective trial on **supportive-expressive group therapy**	Metastatic breast cancer	*N* = 125 (Control *N* = 61; Intervention *N* = 64)	>1 year (14 years follow-up)	**OS** <2.6 months (*p* = 0.73)
Kissane et al. 2007 [13]	Randomized controlled trial on **supportive-expressive group therapy**	Metastatic breast cancer (stage IV)	*N* = 227 (Control *N* = 80; Intervention *N* = 147)	>1 year (2 years follow-up)	**OS** >5.7 months (*p* = 0.60)
					**EORTC QoL C-30**
					Social functioning scale *F* = 4.56 (*p* = 0.03), Impact of Event Scale *F* = 4.61 (*p* = 0.04)
					**Mini-MAC**
					helpless/hopelessness *F* = 4.89 (*p* = 0.03)
Von Minckwitz et al. 2012 [11]	Randomized clinical trial on neoadjuvant therapy with or without **bevacizumab**	Non-metastatic HER2-negative breast cancer	*N* = 1948 (Control *N* = 969; Intervention *N* = 956)	2.5 years	**pCR** >4 % (*p* = 0.04)
Bear et al. 2012 [10]	Randomized clinical trial on neoadjuvant therapy with or without **bevacizumab**	Non-metastatic HER2-negative breast cancer	*N* = 1206 (Control *N* = 596; Intervention *N* = 595)	2.5 years	**pCR** >6.3 % (*p* = 0.02)
Andersen et al. 2008 [14]	Randomized clinical trial on **psychological intervention**	Non-metastatic breast cancer (stage IIA, IIIA or IIIB)	*N* = 227 (Control *N* = 113; Intervention *N* = 114)	1 year (11 years follow-up)	**OS** >1.3 years (*p* = 0.016)
Kissane et al. 2004 [15]	Randomized controlled trial on **cognitive-existential group therapy**	Non-metastatic breast cancer (stage I or II)	*N* = 303 (Control *N* = 149; Intervention *N* = 154)	2 years (5 years follow-up)	**OS** <3.5 months (*p* = 0.31)

Furthermore, we decided to include three meta-analyses on the association between depression and survival [36–38], and other empirical and theoretical studies on Quality of Life of patients, as cancer diagnosis and related treatments have a major negative impact on patients and the measuring of subjective factors allows a unified evaluation of both, targeted therapies and psychological interventions.

### Bevacizumab - a targeted drug for breast cancer

The strategy of new cancer drugs consists in modifying the deregulated intracellular pathways of cancer cells by reprogramming the cell circuit and suppressing, among different biological processes, the acquired neoplastic growth caused by the sprouting of new vessels from existing ones, a biological dynamic also known as angiogenesis [39]. The hypothesis that cancer cells would respond to the pharmacological modification of mutated pathways is based on the notion that intracellular pathways mimic electronic integrated circuits. According to this concept, cancer is a derangement of the integrated circuit of the cell and, therefore, similar to electronic circuits, intracellular pathways should respond to a precisely defined set of rules. Anticancer drugs can then be used to modify the mutated pathways [40, 41].

In the last decade targeted therapy for breast cancer overexpressing the Human Epidermal Growth Factor Receptor 2 (HER2) has been anti-HER2 agents which improves the prognosis of early-stage breast cancer [42]. In advanced and metastatic breast cancer most of the patients become resistant to anti-HER2 drugs; therefore, a new therapeutic strategy was sought in these clinical circumstances [43]. A new class of drugs has been developed to inhibit Vascular Endothelial Growth Factor (VEGF), a diffusible protein produced by tumor cells, that induces blood vessels formation [44]. The inhibition of VEGF and consequently blood vessels formation is achieved by using anti-VEGF monoclonal antibodies, now available on the market as bevacizumab (Avastin) [45]. As manufacturers are under increasing pressure to demonstrate the high clinical value of new costly therapeutics such as bevacizumab, the use of biomarkers to target prospective respondent cancer patients or to exclude those with a low probability of response is a powerful method to boost efficacy and reduce the wastage of resources [46]. This “selection principle” has been trying to be implemented through the use of molecular biomarkers in order to achieve personalised cancer treatment. The great disadvantage for using the targeted drug bevacizumab is that at present no biomarker is available to find the patient subgroup that will benefit from its inclusion as a therapy [47].

Because of the unique role of angiogenesis in cancer progression and the rationale of using anti-VEGF drugs in advanced cancer bevacizumab was approved, through a shortened approval, for the first line treatment of metastatic breast cancer. The shortened process for approval of drugs addressing serious or life threatening diseases is guaranteed by FDA and EMA since 1992 [48]. This process was implemented in order to respond to the increase of cancer incidence and to the needs of patients not responding to the available therapeutics. In this accelerated approval, the endpoints utilized in the normal procedure have been replaced by surrogate endpoints. In the case of drugs for cancer treatments, prolongation of life, measured by overall survival OS, has been substituted by progression-free survival (PFS).

Bevacizumab was approved on the basis of a clinical trial in HER2-negative metastatic breast cancer patients conducted by Miller et al. that showed a benefit of 5.9 months in PFS (*p* < 0.001) by comparing 368 patients receiving chemotherapy with bevacizumab and 354 patients treated with chemotherapy only [9]. The significantly prolonged PFS did not correspond to a significant increase in OS (*p* < 0.16) and also subsequent studies failed to show an OS advantage in metastatic breast cancer while invariably adding serious side effects, in particular hypertension. The surrogate end-point PFS is often not linked with the golden end-point OS, conferring to PFS a limited clinical value [49]. The accelerated approval was not converted into a regular one and in 2010, after three years from its approval, the indication for the use of bevacizumab for metastatic breast cancer patients was revoked by the FDA [50, 51] , although still maintained by EMA [52]. Indeed, for the shortened approval of drugs for metastatic cancer pharmaceutical companies must first supply single-arm trials with PFS as main end-point, then confirmatory post-approval trials must be supplied. If there are no evidences for an increase of OS of patients the indication for that specific use is removed from the label.

‘HER2 positive’ non-metastatic breast cancer patients are eligible for anti HER2 drugs hence, on the basis of the partial response of ‘HER2 negative’ metastatic breast cancer to bevacizumab, its use was studied in the latter category of patients. Early-stage cancer patients are more likely to respond to targeted therapies as drug resistance develops at a late stage. Bevacizumab was added to neoadjuvant chemotherapy and the rate of pathological complete response (pCR) was the main end-point utilized in two studies evaluating the drug. In the study conducted by von Minckwitz et al., the addition of bevacizumab resulted in a moderate increase in breast pathological complete response (pCR) from 20.6 to 24.6 % in which 956 patients who receiving bevacizumab and chemotherapy were compared with 969 patients receiving only chemotherapy (pCR >4 %; *p* = 0.04) [11]. In another study conducted by Bear et al. the addition of bevacizumab resulted in an increase in pCR from 28.2 to 34.5 % by comparing 591 patients with and without bevacizumab (pCR >6.3 %; *p* = 0.02) [10]. When breast and lymphonodes pCR was assessed, no significant increase was found in both the studies. Bevacizumab added its toxic effects to the toxicity of chemotherapy, such as hypertension and ventricular dysfunction. Because of the short observational time, as stated by the authors, it is not yet clear whether the neoadjuvant effect of bevacizumab will translate into a PFS advantage or into a more relevant OS increase.

### Psycho-oncological intervention in breast cancer patients

Cancer patients are increasingly demanding that attention must be given not only to the extension of life but also to its quality related to treatment. The patients’ pressure of considering non biological variables, together with professionals devoted to psycho-oncology are the driving force to encourage the funding of psychosocial studies. Furthermore, lately in USA, cancer centers will be required to implement screening programs for psychosocial distress, enhancing the quality of cancer care and improving health outcomes [53]. The field of psycho-oncology is interested in two major dimensions of cancer: (i) the psychological responses of patients, health care providers and relatives to the disease, and (ii) the psychological, behavioral and social factors that may influence cancer morbidity and mortality. Among the objectives of psycho-oncology are:

- the exploration of the impact of psychological, social and behavioral factors on survival;
- the encouragement of patients’ QoL measurements as an outcome variable;
- the support of broad treatment goals which include patients well-being.

Often oncologists do not consider emotional distress of cancer patients as part of cancer treatment [54]. Psycho-oncological care is based on effective communication between patients and health professionals, where effective communication is defined as “fostering healing relationships, exchanging information, responding to emotions, managing uncertainty, making decisions and enabling self-management” (Epstein and Street 2007 in [54]).

The psychological aspect of cancer patients has been described as “distress” that includes everything from fear, worry, and sadness to disabling problems such as clinical depression, generalized anxiety and existential crisis. Cancer patients need support to cope with their treatments which in combination to the disease give rise to detrimental effects which often require much more attention than the disease itself. Social problems must be addressed by intervening on the patients’ lifestyle such as physical activity and change of diet with the aim of improving physical functioning, while psychotherapy and counseling effectively deal with distress and consequently with QoL. Several studies showed that these interventions can have an impact on survival [55].

Great interest for psycho-oncology arose after the publication in 1989 of a study, selected through our PubMed search, in which OS of 86 women with metastatic breast cancer increased by 17.7 months after enrolment in Supported Expressive Group Therapy (SEGT) [12]. The intent of SEGT is to build new bonds of social support, encourage expression of emotions, deal with fears of dying, help restructure life priorities, improve communication with family members and healthcare professionals, and enhance control of pain and anxiety. The initial favorable outcome was never reproduced and a replica of the study performed later, in 2007, resulted in better OS in 125 metastatic breast cancer patients not receiving group psychotherapy. The treatment period lasted one year but patients were encouraged to remain in contact with the group for the entire time of the assessment that lasted 14 years. Median survival was 30.7 months for the treatment group composed of 64 patients and 33.3 months for the control group composed of 61 patients (OS <2.6 months; *p* = 0.73), but improvement of patients’ well-being was not measured [33].

The second randomized clinical trial for metastatic breast cancer patients by Kissane et al. assessed SEGT in 227 women, diagnosed with stage IV breast cancer, 80 randomized to the control group and 147 randomized to the intervention group. Group therapy consisting of weekly 90-minutes sessions of SEGT lasted one year with a follow-up of 2 years [13]. The model of psychological intervention proposed by Kissane et al. was similar to the one by Spiegel et al.. The trial assessed not only OS of patients, but contrary to the study by Spiegel et al. it showed the effects of SEGT on depression and other Quality of Life measures. The OS did not increase signicantly through SEGT (median survival 24.0 months in SEGT and 18.3 in controls, *p* = 0.60). Several psychosocial well-being measures improved in women receiving SEGT. Specifically, the clinical trial showed the manner in which women in groups sustain humor, creativity, and sense of purpose in their lives despite progressive illness and frailty as they approached their death. Significant improvement in women receiving SEGT occurred in the EORTC (European Organization for Research and Treatment of Cancer) QoL C-30, in the Social Functioning Scale (*F* = 4.56; *p* = 0.03) and in Impact of Event Scale (*F* = 4.61; *p* = 0.04) only for women with a baseline diagnosis of depression. Better attitudinal coping was evident in women receiving SEGT through reduction in scores on the helplessness-hopelessness subscale (*F* = 4.89; *p* = 0.03) of the Mini-MAC (a 29-item questionnaire on Mental Adjustement of Cancer).

In non-metastatic breast cancer patients we selected two studies [14, 15]. The first one by Andersen et al. tested the hypothesis that bio-behavioural or stress related factors are associated with poor survival in women with stage IIA, IIIA or IIIB breast carcinoma. Tumor recurrence and survival were measured in 227 randomly selected women, 114 assigned to intervention and 113 to assessment only [14]. Observational time ranged from 7 to 13 years, with 11 years median follow-up. Psycho-oncological support consisted of psychological counseling, family support, and problem solving counseling. The intervention also aimed at improving health behavior through changing dietary habits, smoking cessation, increasing daily physical exercise, progressive muscle relaxation, support in finding ways to cope with side effects resulting from treatments such as nausea, and adherence to medical treatment and follow-up. The long observational time allowed the calculation of the median survival time, which was 4.8 years for women in the assessment only arm and 6.1 years for women receiving psycho-oncological support (OS >1.3 years; *p* = 0.016). The median time to recurrence was 2.2 years for assessment only arm and 2.8 years for intervention arm [14]. Further analysis of the patients who had cancer recurrence showed that those receiving psycho-oncological interventions had a lower risk of death from cancer, surviving 7 months longer than those not receiving distress reduction support with patients receiving intervention for distress reduction reporting at 12 months a decline in mood disturbance (Hazard Ration 0.982; *p* = 0.022). Mood disturbance was assessed with The Profile of Mood States (POMS), which is a score of five scales, anxiety, depression, anger, fatigue and confusion [35].

The second study we selected in non-metastatic breast cancer patients by Kissane et al. assessed the effects of cognitive-existential group therapy (CEGT) [15]. The intervention was administered to 154 breast cancer patients and compared with 149 patients receiving only adjuvant chemotherapy (all the patients had stage I or stage II breast cancer). The women in the intervention group attended 20 weekly sessions lasting 90 min. The six main goals of CEGT are promoting a supportive environment, facilitating grief, reframing negative thinking, enhancing coping and problem solving, fostering hope, and setting priorities for the future. Patients receiving psychological intervention had a shorter survival of 3.6 months (*p* = 0.31). Although reduced anxiety was detected in women receiving group therapy, survival was significantly associated with tumor histology and node status. Specifically, women receiving group therapy were shown to have reduced anxiety (*p* = 0.05) and a trend towards improved family functioning (*p* = 0.07). Also important, women receiving CEGT reported greater satisfaction with their therapy and increased knowledge about cancer and its treatment (*p* < 0.001) [15].

### Depression and survival – meta-analyses and prospective mechanisms

The effect of emotional distress/depression on survival has been reported in three meta-analses. The first meta-analysis included 157 studies and found that depression was associated with cancer incidence (29 % elevation in HR), survival (8 % elevation in HR) and mortality (34 % elevation in HR) [36]. In a second meta-analysis depression, as measured by Hospital Anxiety and Depression Scale (HADS), was associated with 25 % higher mortality rate after pooling 25 studies for a total of 9417 patients [38]. Another meta-analysis confirmed that depression was associated with a 19 % elevated mortality rate in cancer patients irrespective of the severity of cancer stage [37].

Several possible plausible hypotheses have been proposed to explain the improved survival in cancer patients by distress reduction. The first and most studied mechanism is the reduction of circulating catecholamines, the stress hormones [56]. These hormones compromise cellular immunity by impairing natural killer cells in both, the tumour microenvironment and the peripheral blood; furthermore catecholamines decrease the production of T cell [57]. Weekly sessions of psycho-oncological intervention lasting four months, as described above, has been shown to enhance immune response by increasing T cell proliferation in the adjuvant setting of non-metastatic breast cancer women [34, 57]. Interestingly, bio-behavioural factors are interconnected with the angiogenesis’ pathway, which is the target of bevacizumab. Specifically, depression and loneliness are associated with a higher serum level of VEGF in patients with colon cancer [58], and in patients with colorectal cancer subjected to tumour resection, postoperative serum level of VEGF was correlated with global QoL and cancer related concerns [59]. Moreover, stress response and depression involve secretion of interlukin-6 (IL-6), a pleiotropic, inflammatory cytokine that is also involved in t umour angiogenesis and invasion [56]. It is likely that the release of VEGF, that influences tumour’s vascularization and angiogenesis, is modulated by the stress hormones, as in vitro studies have shown that norepinephrine, a stress hormone, stimulates the production of the angiogenic factor VEGF through a β-adrenergic receptor [58]. Possibly, psycho-oncological interventions reduce the circulating level of VEGF by blocking its release induced by the stress hormones. This biological effect may justify the observed diminished cancer aggressiveness and progression after psycho-oncological interventions.

## Discussion

Personalized medicine for cancer patients through the use of targeted drugs is a promising new therapeutic approach with a rationale of administering drugs that block or inhibit selective molecules critical for cancer development. Targeted drugs are modeled on receptors, signaling pathways and growth factors relevant to cancer cells growth. However, due to the fact that metastatic cancer is a life threatening disease, the fast approval of these drugs has been invoked as an ethical imperative [60]. One of the major obstacles for the health of cancer patients consists in the use of medical treatments by fragmenting the patients into the smallest biological elements [41] where psychological and social aspects of patients are eclipsed. It is important to emphasize that patients’ psycho-oncological aspects have a direct connection with the biological status of patients – as it has been shown psycho-oncological therapy and relaxation interventions may modify physiological stress parameters, biological processes seldom studied in conjunction with psycho-oncological interventions [61]. When administering new drugs to cancer patients, it is first and foremost important to consider a balance between the efficacy of the drug and its side effects. The evaluation of this balance is achieved by QoL measures [62]. Indeed, the very modest benefits of Bevacizumab for both, metastatic and non-metastatic breast cancer patients may be outweighed by the impact of side effects and hence on QoL.

A diagnosis of cancer and the therapies that follow have an impact not only on the physical well-being but also on the social and emotional well-being of patients. Social and emotional well-being is part of the QoL measures. Quality of Life is a complex amalgam of factors divided into different domains; it is indeed a very subjective notion. Nevertheless, there has been an extensive research to quantify subjective factors as evidence for changes in patients’ QoL. By addressing these domains, psychological interventions has obtained, in non-metastatic breast cancer women, an extension of survival [14], possibly through an effect on stress hormones, whereas psychologic interventions on metastatic breast cancer patients have ameliorated their social and psychological status [13]. The European Organization for Research and Treatment of Cancer (EORTC) has developed a scientific method for measuring cancer patients’ QoL. The Health related Quality of Life QLQ-C30 questionnaire is a validated questionnaire and it has been translated in more than 81 languages. The EORTC QLQ-C30 is one of the most commonly used instruments, consisting of 30 questions comprising 3 symptom scales (Pain, Fatigue, and Nausea/Vomiting), 6 single-items (Dyspnea, Insomnia, Appetite loss, Diarrhea, Costipation, and Financial difficulties), 5 functioning scales (Physical, Role, Emotional, Cognitive, and Social), and two questions referring to the overall health and quality of life [63].

### Quality of Life measures and targeted drugs – an ethical appraisal

According to our knowledge physicians mostly rely on their subjective observations of cancer patients’ symptoms and QoL measures are not commonly used for treatment with targeted therapies, despite they are increasingly recognized as a valuable tool to measure effectiveness of cancer therapy [64], and in spite of the diagnosis of cancer and related treatment that have an impact on QoL, depending on the individual’s perception through the personal situation. Patients with cancer are challenged by finiteness and death, so that the opportunity of personalized medicine, notably after a long cancer treatment course, might seem very inviting.

The variables measured through the EORTC QLQ-C30 were found to be significant prognostic indicators of survival in different cancer patients although evidence is controversial and not definitive [65, 66]. Because of the association between QLQ-C30 measures and survival this questionnaire is included as a valuable non-conventional endpoint in clinical trials [67]. Metastatic breast cancer patients have a modest median survival and in clinical trials, when survival benefit is presumably limited, QoL assessment is particularly helpful and suitable. Drug ability to extend life is viewed by many oncologists and by medicine agencies as the gold standard in assessing cancer drugs effectiveness, although for individual management of patients QoL measures are considered the most appropriate endpoint [68, 69]. In both, the psycho-oncology and the bevacizumab clinical trials on metastatic breast cancer patients the main endpoint was survival and both interventions did not attain any increase of it. The lack of QoL measures prevented the evaluation of emotional, social or functional patients’ improvement with psycho-oncological interventions. Moreover, as QoL data and psychological distress both predict OS in breast cancer patients (see the meta-analyses above), their availability in reported trials would have added evidence of the causes of the unsuccessful extension of survival. The information given to and received from cancer patients can be based on the scientifically understanding of their experiences on cancer treatments gained through QoL measures; this can not only improve the patient-doctor relationship through the inclusion of practitioners prone to develop a bi-directional communication with patients, but can also engage actively patients in their care. More relevant, the QoL questionnaires are instruments to promote the investigation between length and quality of survival [65], where QoL measures open the realm to patients’ values and expressions of their experiences. These subjective factors are assessed through scientific methods of enquiry and represent one of the bridges between a medicine intended as art and another one intended as science. The objective knowledge of psychosocial aspects of medicine – i.e. Quality of Life measures – might be instrumentally used to modulate the pathophysiological effects of biological intervention – i.e. targeted drugs as bevacizumab. Then, it can be ethically advisable to use both the approaches, psychological interventions and biological targeted drugs on those patients for which other drugs are not available. Moreover, when the patient wants to participate in the decision making about her future treatments, namely when she claims her autonomy, it is central to activate policies to translate the information contained in studies like this one, and focusing on treatments’ outcomes, in a language able to convey information on specific interventions and available options [70]. From an ethical perspective protection of vulnerable patients is of paramount importance as their decisions on future treatments might easily be bent by clinicians – advanced-stage cancer patients develop hope in medicines alongside trust in professional, overlooking doubts regarding drugs, trials and clinicians [71]. Using psychological interventions and measures on components of patients’ quality of life, it might be possible to mitigate side-effects of cancer therapeutics. This comprehensive, integrate approach for cancer patients not only goes towards more effective treatments but it also represents a unification between a medicine made of ahistorical and unnatural molecules and a medicine rooted in humanistic values, articulated to satisfy needs of patients [3].

The use of a standardized questionnaire, like the EORTC QoL C-30, across different countries and sites seems advisable. In the present study we found very difficult to compare effects derived from psychological interventions on cancer patients as the endpoints were homogeneous – i.e. only the study by Kissane et al. on metastatic breast cancer patients reported measures zoperated through the EORTC QoL C-30 [13]. A prospective agreement on commune measures of patients’ quality of life might be very useful in order to integrate a QoL endpoint in clinical trials and approval processes.

## Limitations

This study has limitations derived from method utilized to gather the literature and derived from quality of data of the article analysed. Articles selected to scrutinize the impact of bevacizumab in early-stage non-methastatic breast cancer patients have been chosen without a specific search strategy. Despite most of the articles on this topic report negative and not confirmatory results, an extended analysis might be of help for those wanting to develop policies on the use of bevacizumab for patients with early-stage breast cancer. For those wanting to evaluate the controversial use of bevacizumab on endocrine refractory or resistant metastatic breast cancer patients, a Cochraine review done by Wagner et al. is worth to be considered [72]. The search strategy to retrieve the articles scrutinizing effects of psychological interventions for breast cancer patients was limited to a single database. Few more studies might have gained by using other databases and auxiliary search strategies.

A second major limitation was due to the quality of data retrieved and reported as not enriched with details such as cancer stage and revelation index. These data were present only in few retrieved articles. A systematic review produced by Casellas-Grau et al. and a Cochrane meta-analyses produced by Jassim et al., both on psychological interventions in breast cancer might very useful to have at hand sound empirical evidences [73, 74].

## Conclusion

The QoL measures are considered helpful in choosing the right therapy for cancer patients, as psychological distress has an impact on perceived health. It would be therefore of pivotal interest for both, psycho-oncologists and clinical oncologists to take into consideration a tool to measure patients well-being in order to face the extremely difficult management of metastatic and non-metastatic cancer patients [75]. From a prospective point of view, if we desire to implement personalized medicine through the use of targeted drugs, it is important to compare and consider not only the molecular aspects of cancer but also patients’ psychosocial health, where communication and humanistic values are taken in consideration. The incorporation of the self-reported QoL questionnaires in clinical trials can pave the road for not only the amelioration of patients’ symptoms but also for an extension of their survival. The comparison between the two different approaches through a commonly used questionnaire can help in developing a more comprehensive and effective use of targeted drugs that in turn could lead towards the validation of useful cancer biomarkers and endpoints.

## Abbreviations

CEGT: Cognitive Existential Group Therapy
EMA: European Medicines Agency
EORTC: European Organisation for Research and Treatment of Cancer
FDA: Food and Drug Administration
HADS: Hospital Anxiety and Depression Scale
HER2: Human Epidermal Growth Factor Receptor 2
Mini-MAC: a 29-item questionnaire on Mental Adjustment of Cancer
OS: Overall Survival
pCR: Pathological complete response
PFS: Progression Free Survival
POMS: Profile of Mood States
PROs: Patient Reported Outcomes
QoL: Quality of Life
SEGT: Supported Expressive Group Therapy
VEGF: vascular endothelial growth factor
